# Pore Narrowing of Mesoporous Silica Materials

**DOI:** 10.3390/ma6020570

**Published:** 2013-02-20

**Authors:** Frederik Goethals, Elisabeth Levrau, Els De Canck, Mikhail R. Baklanov, Christophe Detavernier, Isabel Van Driessche, Pascal Van Der Voort

**Affiliations:** 1 Inorganic and Physical Chemistry, Ghent University, Krijgslaan 281 (S3), Gent 9000, Belgium; E-Mails: frederik.goethals@ugent.be (F.G.); els.decanck@ugent.be (E.D.C.); isabel.vandriessche@ugent.be (I.V.D.); 2 Solid State Sciences, Ghent University, Krijgslaan 281 (S1), Gent 9000, Belgium; E-Mails: elisabeth.levrau@ugent.be (E.L.); christophe.detavernier@ugent.be (C.D.); 3 Imec (Interuniversity Microelectronics Centre), Kapeldreef 175, Heverlee 3001, Belgium; E-Mail: baklanov@imec.be (M.R.B.)

**Keywords:** pore sealing, MCM-41, bridged organosilanes

## Abstract

To use mesoporous silicas as low-k materials, the pore entrances must be really small to avoid diffusion of metals that can increase the dielectric constant of the low-k dielectric. In this paper we present a new method to narrow the pores of mesoporous materials through grafting of a cyclic-bridged organosilane precursor. As mesoporous material, the well-studied MCM-41 powder was selected to allow an easy characterization of the grafting reactions. Firstly, the successful grafting of the cyclic-bridged organosilane precursor on MCM-41 is presented. Secondly, it is demonstrated that pore narrowing can be obtained without losing porosity by removing the porogen template after grafting. The remaining silanols in the pores can then be end-capped with hexamethyl disilazane (HMDS) to make the material completely hydrophobic. Finally, we applied the pore narrowing method on organosilica films to prove that this method is also successful on existing low-k materials.

## 1. Introduction

Nanoporous organosilica materials can be used as insulating (low-k) materials in microelectronic devices because the presence of air-filled pores lowers the dielectric constant significantly [1]. Insulators with lower k-values decrease RC-delay and power consumption of microelectronic devices [2]. However, during integration of such materials in integrated circuits, several problems can occur when using porous materials [3]. Firstly, chemical products that are used during processing and water can diffuse into the porous material thereby increasing the dielectric constant of the low-k material. Further, metal ions from the wires can move in the pores during polishing processes or via electromigration when the device is in use, leading to short circuits and high leakage currents [4].

Dealing with very small (<1 nm) or not interconnected pores, the previous described issues are negligible. However, as the dielectric constant approaches values below 2.2, pore sizes tend to increase and the pores are highly interconnected [5]. Therefore, a pore narrowing step or pore sealing step is necessary.

Pores up to 2 nm can be sealed with plasma treatments [6,7], chemical vapor deposition (CVD) [8] or atomic layer deposition (ALD) [9]. However, when the pore sizes are larger than 2 nm these methods are no longer efficient. Plasma treatments can damage the mesoporous low-k films [10], while CVD and ALD precursors have a high k-value or diffuse into the low-k material resulting in pore filling instead of pore sealing, thereby also increasing the overall k-value [3].

Therefore a pre-sealing step that narrows the pores on the surface is required. The requirement for this pre-sealing step is that it does not significantly increase the dielectric constant of the low-k material. Therefore the sealant molecules itself must have a low dielectric constant and diffusion of the sealants into the porous low-k layer has to be avoided [11,12,13].

An interesting method to achieve this is a thermal treatment of mesoporous silicas and organosilicas that have narrow passages. The thermal treatment closes the narrow passages due to framework shrinkage resulting in ordered arrays of closed mesopores [14,15,16].

Recently, we reported a possible pre-sealing method by depositing a non porous layer on top of a mesoporous film [17]. This method involved the molecular self-assembling of cyclic carbon-bridged organosilane precursors which allows the formation of intermediate fragments with molecular sizes exceeding pore sizes of 3 nm. Spin-coating of these fragments on top of porous films therefore allowed efficient sealing of mesoporous low-k materials.

**Figure 1:**
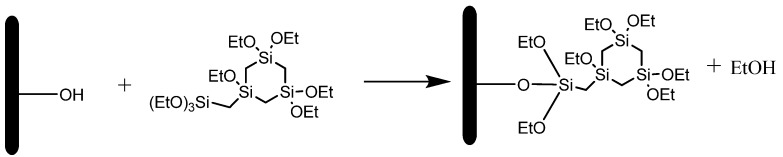
Grafting cyclic bridged organosilane precursor on MCM-41.

In this paper, we report another method to narrow the pore sizes without losing the porosity of the mesoporous material. This method is based on a grafting reaction on a mesoporous material with a cyclic-bridged organosilane precursor (presented in Figure 1). A huge advantage of this precursor is its intrinsic low k-value (≈3.5) owing to the high amount of low polarisable organic groups [18].

Firstly, it is shown that the grafting on the porous material is successful. In a second step, it is presented that grafting before surfactant removal prevents diffusion of the precursor molecules into the porous material. As a proof of concept, the experiments were firstly performed on MCM-41 powders. MCM-41 powders are mesoporous and consist of a pure silica matrix [19] making it easier to monitor the grafting with the organosilane precursor. Finally, the method was applied on thin organosilica films.

## 2. Experimental Section

### 2.1. Chemicals

Chloromethyltriethoxysilane, 1,2-bis(triethoxysilyl)ethane (BTESE), tetraethylorthosilicate (TEOS) and hexamethyl disilazane (HMDS) were purchased from ABCR, hydrochloric acid (HCl, 37%), tetrahydrofuran (THF), pentane and absolute ethanol were obtained from Fiers, and cetyl trimethylammonium chloride (CTAC, 25%) and cetyl trimethylammonium bromide (CTAB) were purchased from Aldrich. All materials were used as received.

### 2.2. Synthesis

**Figure 2:**
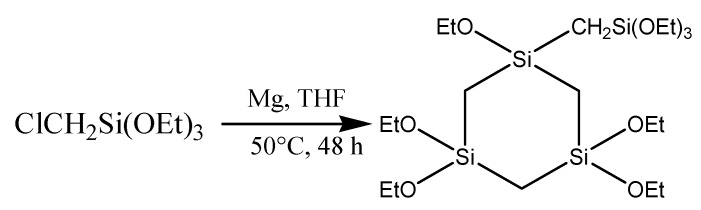
Synthesis of cyclic-bridged organosilane precursor.

Preparation of the cyclic-bridged organosilane precursor was based on a method of Brondani* et al.* [20]. A solution of 70 mL 0.5 wt % FeCl_3_ in dry THF was added to 7 g Mg turnings and stirred until a grey colored mixture was visible. This mixture was kept under an inert atmosphere. Then, a solution of 100 mL 14.2 vol % chloromethyltriethoxysilane in dry THF was rapidly added to the mixture and stirred for 48 h at 50 °C. The mixture was filtered off and the solvent was removed from the filtrate. Pentane was added to the residue and this mixture was also filtered. The remaining oil consists of cyclic carbon-bridged organosilanes. The applied cyclic silane precursor was separated by removing undesired precursors via distillation. The remaining oil consists almost completely of the required cyclic precursor. This was confirmed by an in depth nuclear magnetic resonance (NMR) study which is provided as supplementary information. A schematic synthesis procedure for this precursor is given in Figure 2.

#### 2.2.1. Synthesis of MCM-41

The synthesis of MCM-41 was based on the method of Mortera* et al.* [21] CTAB, H_2_O, EtOH and NH_4_OH were mixed and stirred for 1 h at room temperature. Next, TEOS was added and the mixture was left for reaction for 1 h. The total molar composition was: TEOS: 1; CTAB: 0.3; H_2_O: 144; EtOH: 58; NH_4_OH: 8. The precipitated powder was filtered and washed with water. The obtained powder was then separated in two parts. From one part the surfactant was removed by calcination at 550 °C, while for the other part the surfactant was left inside.

#### 2.2.2. Synthesis of Organosilica Films

Exactly 3.2 g 25 wt % CTAC was dissolved in 10 mL ethanol and 0.7 mL (1 M) HCl. Then, 1 mL of BTESE was added to the solution and this was aged for 1 day. The solution was then spin-coated on a Si wafer at a rate of 5000 rpm. The surfactant was removed by treating the film at 400 °C for 5 h.

#### 2.2.3. Grafting on MCM-41

To attach the cyclic organosilane precursor on MCM-41, 0.3 g of MCM material with or without surfactant was first dried under vacuum at 150 °C. Then, 0.9 mL of the organosilane precursor was added and refluxed for 5 h to allow reaction of the molecule with the MCM powder. The powder without surfactant was filtered and the excess amount of unreacted precursor was removed by Soxhlet extraction with pentane. For the powders with surfactant, only filtration and washing with acetone was performed to prevent removal of the surfactant.

#### 2.2.4. Grafting on the Organosilica Films

The films were dried overnight at 90 °C under vacuum. Next, an argon flow was applied and the precursor was added. The temperature was elevated to 130 °C and the system was again put under vacuum to bring the precursor in the gas phase. The precursor was allowed to react for 3 h. Afterwards, the films were rinsed with pentane and the surfactant was thermally removed at 400 °C under nitrogen. Finally, the films were immersed for 3 h in pentane containing HMDS to allow reaction with the HMDS molecules and afterwards dried at 150 °C for 2 h.

### 2.3. Characterization

N_2_ sorption isotherms were measured on a Belsorp-Mini II apparatus at 77 K. The data of the adsorption branch were used to calculate the pore diameter using the BJH method.

Diffuse reflectance infra-red Fourier transform (DRIFT) spectra were obtained on a Thermo 6700 FLEX FTIR/FT-Raman system, equipped with a nitrogen cooled MCT-A detector.

The porosity of the films was determined with ellipsometric porosimetry. Therefore, a spectroscopic ellipsometer Sentech 801 is mounted in a vacuum chamber that can be filled with solvent vapor (toluene) in a controlled way. The pressure of the toluene vapor is raised in steps from the vacuum level up to the saturation pressure. The pressure dependent condensation occurs in the open pores and the refractive index of the sample is changed. The total pore volume is calculated from the change in refractive index at saturation pressure using the Lorentz-Lorenz equation.

(1) $$P=\;\left(\frac{n_{\text{rf}}^{2}-1}{n_{\text{rf}}^{2}+2}-\frac{n_{\text{re}}^{2}-1}{n_{\text{re}}^{2}+2}\right)/\;\left(\frac{n_{\text{ads}}^{2}-1}{n_{\text{ads}}^{2}+2}\right)$$

With *P* the porosity, *n*_re_ the refractive index of the film with empty pores, *n*_rf_ the refractive index of the film with filled pores and *n*_ads_ the refractive index of the solvent.

Water contact angles values were obtained by using a Krüss-DSA 30 Drop Shape Analysis System using the tangent 1 model.

One dimensional (1D) and two dimensional (2D) ^1^H and ^13^C NMR spectra were recorded on a Bruker Avance 300 MHz spectrometer. Chemical shift values (δ) are given in parts per million (ppm) and are referenced to the residual CDCl_3_.

## 3. Results and Discussion

### 3.1. Grafting on MCM-41 without Presence of Surfactant

**Figure 3:**
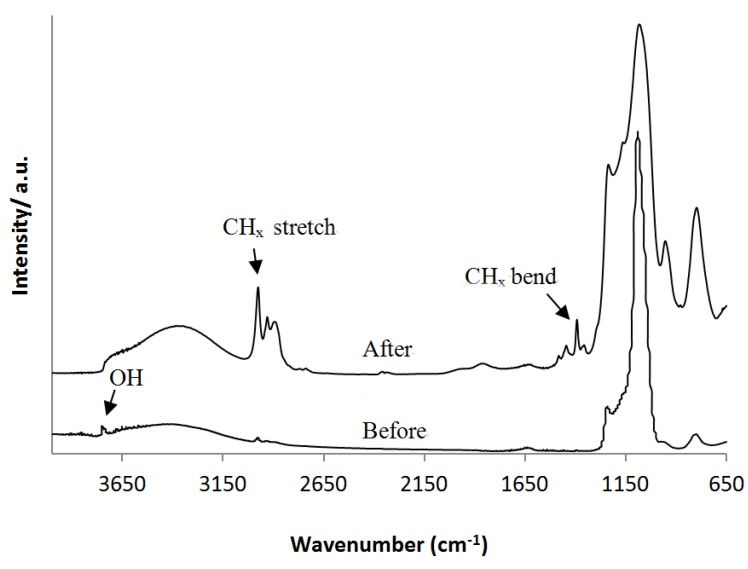
Diffuse reflectance infra-red Fourier transform (DRIFT) spectrum of MCM-41 before and after grafting.

On template-free MCM-41 a grafting is performed according to the reaction presented in Figure 1. The silanols of the MCM material react with the Si centers of the silane precursor and ethanol is released. After the grafting reaction, the material was rinsed several times with pentane and dried at 400 °C under nitrogen to remove remaining precursor molecules. Proof for completion of the reaction is given by DRIFT spectroscopy and N_2_ sorption measurements. Before grafting, the DRIFT spectrum in Figure 3 shows a typical spectrum for MCM materials. At 1150 cm^−1^ the Si–O–Si peak is clearly visible and the peak at 3750 cm^−1^ can be assigned to the free silanol groups. After grafting the peak of the free silanols disappeared and C–H stretch vibrations of the organosilanes are clearly visible around 2970 cm^−1^ as well as C–H bend vibrations around 1400 cm^−1^.

Due to the removal of a large amount of silanol groups by these silane molecules, the material is more hydrophobic. This was observed by pouring the grafted powder in water. The grafted powder floated on the water surface while on the other hand pure (hydrophilic) MCM powder immediately sinks to the bottom.

**Figure 4:**
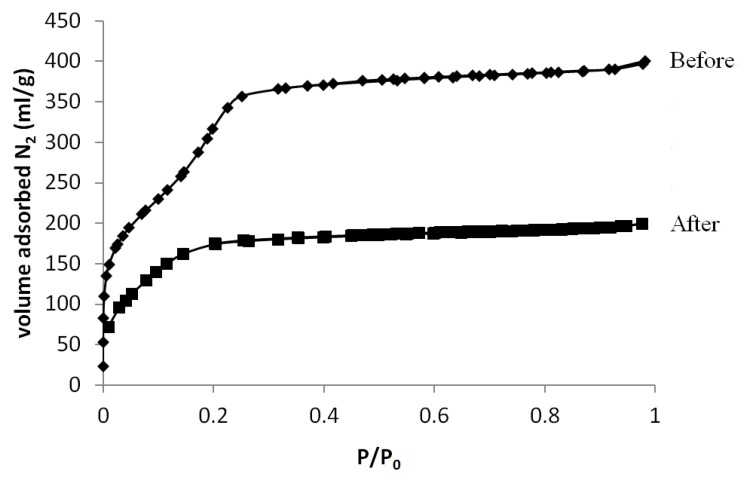
Nitrogen sorption isotherm of MCM-41 before and after grafting.

The N_2_ sorption isotherms before and after grafting are presented in Figure 4. The isotherm before grafting shows a typical type IV isotherm. The pore volume is 0.6 mL/g and the average pore diameter is 2.2 nm. The nitrogen sorption isotherm of the grafted powder gives a type I isotherm, revealing that the material is microporous instead of mesoporous. This can be explained by the fact that the pore diameter of MCM-41 is on the edge between micro- and mesopores. Therefore, grafting on such materials will shift pore dimensions from mesoporous to microporous. The pore volume has decreased from 0.6 mL/g before grafting to 0.30 mL/g after grafting meaning that the grafting molecules could easily diffuse into the porous material and react with the inner silanol groups, thereby reducing the pore size and pore volume. Of course, when applying this method for pore narrowing, reduction in pore volume should be prevented.

### 3.2. Grafting on MCM-41 Where the Surfactant was Initially Left Inside

To avoid pore filling, the grafting reaction was performed on MCM-41 powders where the pores were still filled with the surfactant. After the grafting, the surfactant was thermally removed (at 400 °C under inert atmosphere) and nitrogen adsorption measurements were performed.

**Figure 5:**
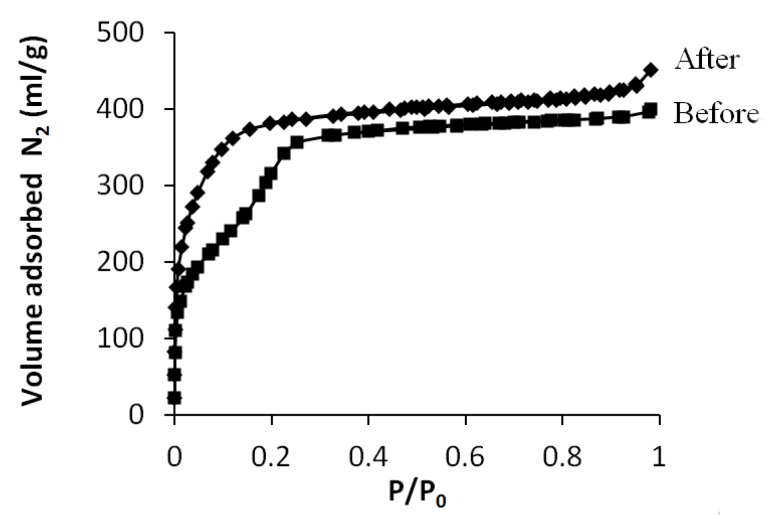
N_2_ sorption isotherms of MCM-41 and MCM-41 after grafting.

When comparing the isotherms in Figure 5 before and after grafting, it can be seen that the total pore volume is similar within the experimental error (0.66 mL/g and 0.62 mL/g respectively), showing that a high porosity is maintained and meaning that grafting mainly took place at the pore entrances.

After the grafting a type I isotherm typical for microporous materials is observed. This can be explained by the fact that besides the intended grafting on the pore entrance, a small amount of grafting molecules diffuses in the surfactant loaded pores anyway and reacts with the silanols inside the pores. As the original pores are already on the edge between micro- and mesopores, this is already sufficient to shift the isotherm from a type IV to a type I. Furthermore, because the original porosity is maintained, this result is beneficial for technological low-k applications as micropores can be efficiently sealed (in contrast to mesoporous materials) by plasma treatments, CVD or ALD [3,22].

The unreacted inner silanol groups are afterwards end-capped with HMDS to make the material completely hydrophobic and therefore avoiding moisture adsorption.

**Figure 6:**
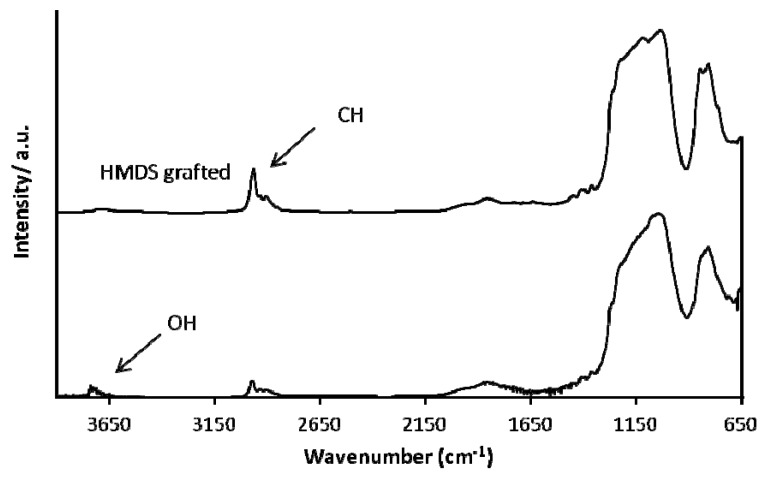
DRIFT spectrum of functionalized MCM-41 before and after hexamethyl disilazane (HMDS) treatment.

The DRIFT spectrum gives evidence that the free silanol groups are end-capped with trimethyl silyl groups (Figure 6). Before the HMDS treatment, the DRIFT spectrum shows CH stretch vibrations peaks around 2950 cm^−1^ related to the grafted cyclic organosilane precursor and OH vibrations at 3750 cm^−1^ from the MCM-41 powder. After the HMDS treatment the silanol peak disappears and the peak at 2960 cm^−1^ increases due to the extra CH_3_ groups. No typical water absorption peaks are observed, confirming that the material is completely hydrophobic.

### 3.3. Grafting on Organosilica Films

Because most of the technological low-k materials consist of an organosilica framework and are processed as thin films [1], we applied the pore narrowing method described in Section 3.2 (grafting before surfactant removal and HMDS treatment) on porous ethylene-bridged organosilica films. Because nitrogen adsorption is no longer accurate for thin films on a substrate, the porosity was determined with ellipsometric porosimetry using toluene as adsorbent [23,24].

**Figure 7:**
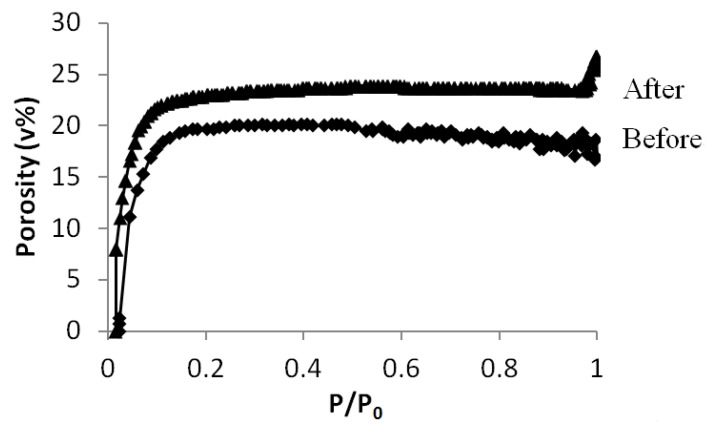
Toluene adsorption isotherms before (1) and after grafting (2) on mesoporous organosilica films followed with HMDS treatment.

**Figure 8:**
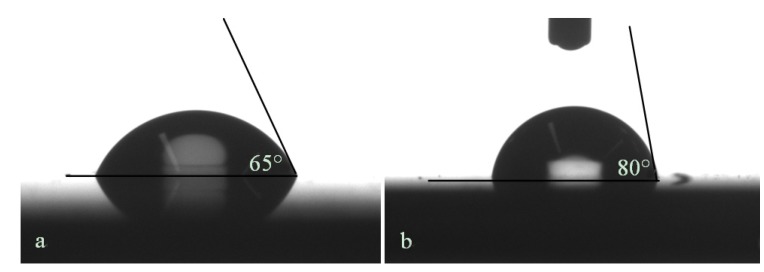
Water contact angle of the organosilica film (**a**) before and (**b**) after grafting.

The resulting toluene adsorption isotherms before and after the grafting are presented in Figure 7. It can be seen that after grafting, the adsorption takes place at lower pressures indicating that the pores are indeed narrowed. Further, the total porosity is slightly higher after grafting. This can be explained by the fact that the material is hydrophobic after grafting preventing water adsorption which is the case for the pristine film. This is also confirmed by the higher water contact angle which was found to be 80° after grafting as compared to 65° before grafting (see Figure 8).

## 4. Conclusions

It is shown that it is possible to graft cyclic bridged organosilane precursors on mesoporous silicas thereby narrowing the pore sizes. While keeping the surfactant inside the material during the grafting, only the silanols at the outer surface and pore entrances are end-capped. With this approach, pore narrowing can be obtained without decreasing the total porosity of the material. The silanols inside the pores can be grafted with HMDS, making them non reactive and hydrophobic.

Transferring this method to thin films showed that it is also possible to narrow the pore openings on the top surface without decreasing the total porosity. Furthermore, the film is more hydrophobic after the grafting with the cyclic carbon-bridged organosilane precursors.

## Supplementary materials

Supplementary File 1
