# Supplementary material for: Pore Narrowing of Mesoporous Silica Materials
Source: Materials (Basel). 2013 Feb 20;6(2):570–9. doi: 10.3390/ma6020570 (PMC5452099; doi:10.3390/ma6020570)
Supplement: Supplementary File 1 [file materials-06-00570-s001.pdf]

Supporting Information: NMR Study of the Substituted Cyclic Organosilane Precursor

The obtained cyclic organosilane oil is studied with one dimensional (1D) ^{1}H (Figure S1) and ^{13}C NMR (Figure S2) spectra complemented with two dimensional (2D) experiments such as homonuclear ^{1}H-{^{1}H} Correlation spectroscopy (COSY) and heteronuclear ^{1}H-{^{13}C} Heteronuclear Single Quantum Correlation (HSQC) and Heteronuclear Multiple Bond Connectivity (HMBC) experiments (not shown). In Figure S1 the ^{1}H resonances at 1.20 ppm and at 3.8 ppm are clearly visible. Based upon their multiplicity, signal integrations and COSY correlations, these resonances can be attributed to the $–CH_2–$ and $–CH_3$ moieties of the ethoxy functionality. Using HSQC and HMBC experiments additional to the ^{13}C spectrum, the carbon atoms can be assigned.

Furthermore a set of broad resonances in the region of 0.3 to −0.01 is observed in the 1D ^{1}H spectrum. They are identified by using the 2D NMR techniques as the protons of the $–CH_2–$ moieties of the ring structure and of the additional substituent $–CH_2Si(OEt)_3$. This is in good agreement with the chemical shift values described in literature [1]. Although rather sharp singlets are expected for these particular protons, a more complex spectrum is observed. This can be explained by a non-equivalent chemical environment of the protons due to a conformational inversion of the six-membered ring.

Figure S1. 1D ^{1}H nuclear magnetic resonance (NMR) spectrum of the cyclic organosilane oil.

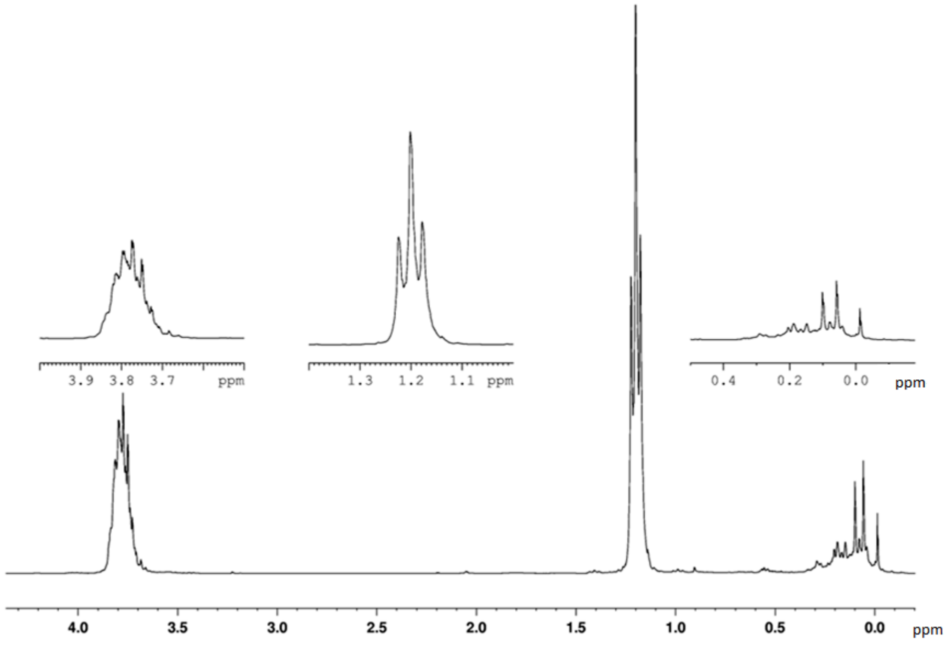

Figure S2. ^{13}C APT NMR spectrum.

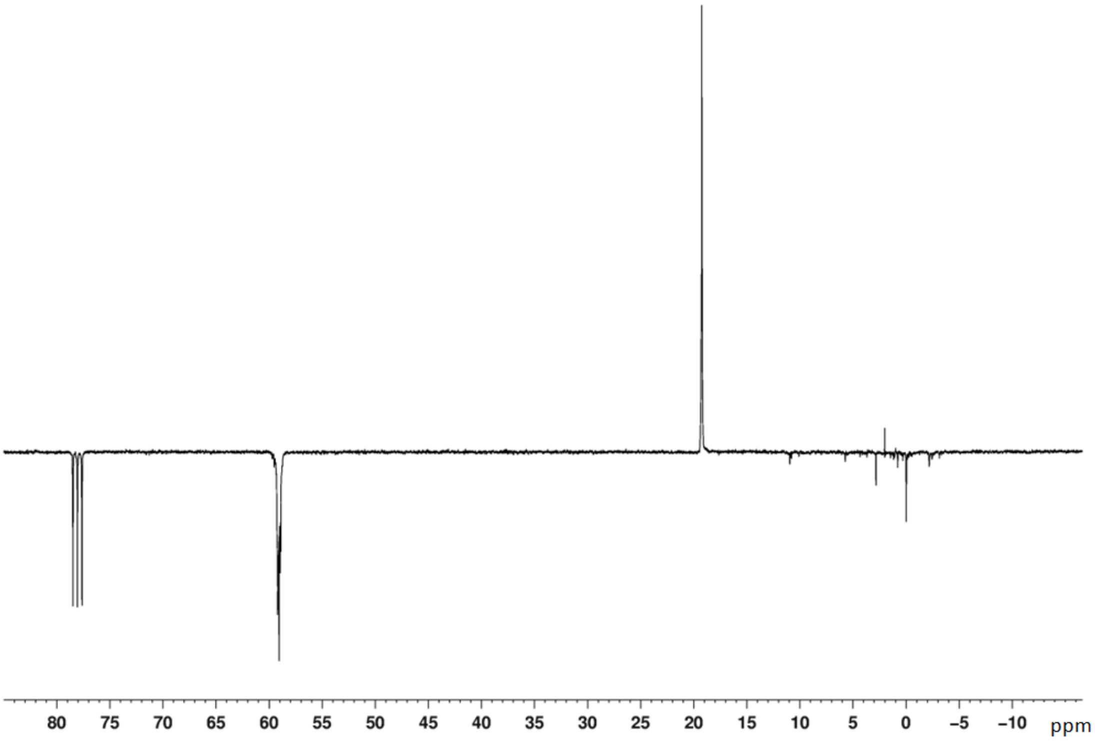

The possibility exists that during synthesis, the cyclic organosilane precursor of Figure 2 in the paper reacts further with the formation of more branched products. Additional $–CH_2–Si(OEt)_3$ are at first sight not observed in the ^1H and ^{13}C NMR spectra and furthermore the signal integrations of the protons do not sustain the presence of more branched compounds in the mixture. Nevertheless, these products can be present in a very small amount and therefore perhaps not detectable. Thus, although it was not possible to completely exclude the presence of more branched silanes, these NMR results confirm that the organosilane oil consists almost completely of the substituted tri-silacyclohexane.

Figure S3. scanning electron microscope (SEM) image of the synthesizedorganosilica thin film.

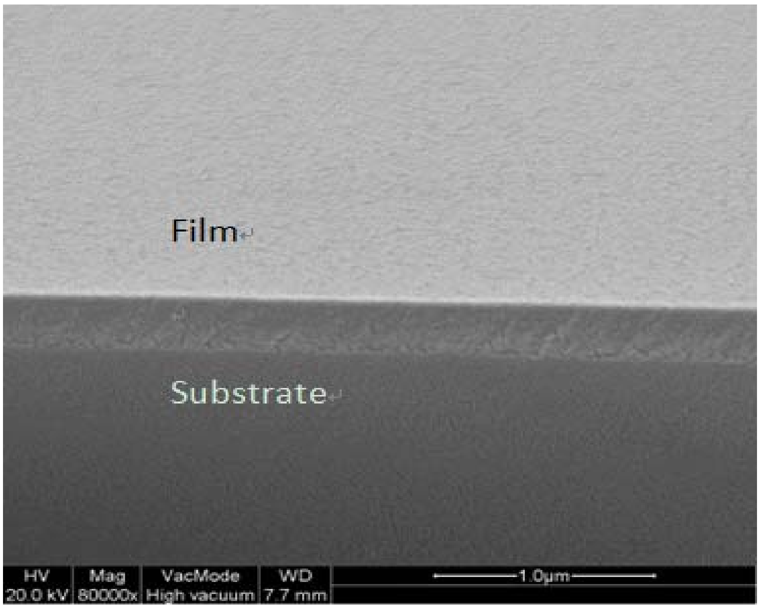

References

1. Brondani, D.J.; Corriu, R.J.P.; Elayoubi, S.; Moreau, J.J.E.; Man, M.W.C. Polyfunctional carbosilanes and organosilicon compounds—Synthesis via grignard reactions. *Tetrahedron Lett.* **1993**, *34*, 2111–2114.

© 2013 by the authors; licensee MDPI, Basel, Switzerland. This article is an open access article distributed under the terms and conditions of the Creative Commons Attribution license (http://creativecommons.org/licenses/by/3.0/).
